# Imaging Biomarkers for HER2-Positive Breast Cancer: Evidence from an Observational Study

**DOI:** 10.3390/jcm14145056

**Published:** 2025-07-17

**Authors:** Sara Boemi, Alessia Pagana, Maria Teresa Bruno

**Affiliations:** 1 Department of Radiology, Fondazione IRCCS Cà Granda, University of Milan, 20122 Milano, Italy; 2 Department of General Surgery and Medical-Surgical Specialties, Gynecological Clinic, University of Catania, 95123 Catania, Italy; alessia.pagana@studium.unict.it (A.P.); mt.bruno@unict.it (M.T.B.)

**Keywords:** breast cancer, HER2, microcalcifications, mammography, logistic regression, breast imaging

## Abstract

**Background**: Mammographic microcalcifications (MCs) are a common early radiological finding in breast cancer, but their significance in relation to molecular subtypes, particularly HER2-positive tumors, remains under investigation. **Objectives**: To evaluate the association between MCs and HER2 status in invasive breast cancer. **Methods**: A retrospective study was conducted on 185 patients treated at a breast unit between 2018 and 2023. Clinical, histological, and molecular data were analyzed. Logistic regression was used to identify independent predictors of MCs. **Results**: MCs were present in 27% of HER2-positive patients and 16.15% of HER2-negative patients (*p* < 0.001). HER2 positivity was the only significant independent predictor (OR = 5.89; 95% CI: 2.42–14.30; *p* < 0.001). Age, breast density, and histology were not associated. **Conclusions**: MCs are significantly associated with HER2 positivity and may serve as an early imaging marker of aggressive disease, supporting the integration of radiologic and molecular diagnostics.

## 1. Introduction

Breast cancer is the most frequently diagnosed malignancy in women globally and is a major cause of female cancer mortality [1,2,3]. The World Health Organization (WHO) stated that breast cancer will cause 670,000 deaths globally in 2022 [4]. It is a global public health problem, as it is expected that by 2040, there will be over 3 million cases and 1 million deaths from breast cancer annually [2]. Despite significant advances in screening, early detection, and personalized therapies, the biological heterogeneity of breast cancers carries important clinical and prognostic implications [5]. The identification of molecular subtypes, including assessment of receptor status (ER, PR, HER2), has revolutionized the diagnostic–therapeutic approach, allowing more accurate stratification of patients and the introduction of targeted therapies [6]. Four molecular subtypes have been identified: (1) luminal A, (2) luminal B, (3) human epidermal growth factor receptor 2 (HER2)-enriched, and (4) triple negative. Luminal tumors are the most common (60–70%) and are characterized by estrogen receptor (ER) expression. Luminal A tumors have the best prognosis among all subtypes, while patients with luminal B tumors have significantly shorter overall and disease-free survival. Both commonly present as irregular masses without mammographically associated calcifications. HER2-positive carcinoma is characterized by overexpression of the human epidermal growth factor receptor 2 (HER2) receptor, which promotes cell proliferation and survival, and from low or absent ER expression [7]. This subtype accounts for about 15–20% of breast carcinomas and is associated with aggressive behavior, high proliferation, increased risk of recurrence, and early metastasis [8,9]. The introduction of anti-HER2 monoclonal antibodies has significantly improved prognosis, making early diagnosis of this subtype even more crucial [10]. HER2+ tumors most commonly present as spiculated masses with pleomorphic calcifications or as calcifications alone. Triple-negative or basal-type carcinomas account for 15% of all invasive breast cancers and are characterized by lack of ER, progesterone receptor (PR), and HER2 expression. Basal-type carcinomas are often high-grade, large at diagnosis, and have high recurrence rates.

Radiologically, mammography is the main screening tool for breast cancer [11]. Microcalcifications (MCs) are among the most common findings observed on mammography and may be the first radiological sign of malignancy [12,13]. However, not all MCs have the same predictive value; those with pleomorphic morphology or linear or segmental distribution are considered suspicious for malignancy [14,15]. The potential association between the presence of microcalcifications and specific molecular subtypes has attracted scientific interest; however, the available literature remains limited and sometimes contradictory.

Some studies have suggested a higher incidence of MCs in HER2-positive breast tumors, hypothesizing a link between tumor morphological characteristics and calcification formation [16,17].

There are numerous comparative studies that have explored the correlation between mammographic microcalcifications and HER2-positive breast cancer.

Lee et al. [18] demonstrated that microcalcifications are significantly associated with HER2-positive tumors, suggesting their potential role as indirect indicators of aggressive tumor biology. O’Grady & Morgan [19] performed a comparative study showing that HER2-positive tumors exhibit a higher frequency of branching calcifications and segmental distribution compared to luminal A or triple-negative subtypes.

Scimeca et al. [20] reported that HER2-positive tumor cells can acquire an osteoblastic phenotype, actively producing hydroxyapatite crystals and contributing to the formation of malignant microcalcifications. Wang X et al. [21] used advanced imaging and showed that linear microcalcifications are significantly more frequent in HER2-positive cancers, with high positive predictive value.

Radiogenomics and multimodal imaging (mammography, DCE-MRI, radiomic ultrasound) seem to offer further insights: calcification patterns associated with HER2 positivity have been correlated with pro-calcification gene expression profiles and angiogenic activity.

In addition, recent literature supports the hypothesis that microcalcifications may act as independent imaging markers of HER2-positive tumors. However, a thorough evaluation by multivariate cohort analysis is lacking.

The aim of this study is to evaluate whether MCs are independent markers associated with HER2 positivity in invasive breast cancer populations. We used multivariate logistic regression models including age, breast density, and histology to determine whether MCs can be adopted in diagnostic pathways for targeted early diagnosis.

## 2. Materials and Methods

A retrospective observational study was conducted at the Senology Center of the Fatebenefratelli Hospital in Milan, based on a consecutive cohort of patients treated for invasive breast cancer between January 2018 and December 2023.

The study population included 283 women with a histologically confirmed diagnosis of invasive breast cancer. Patients diagnosed with carcinoma in situ without an invasive component, synchronous bilateral tumors, local recurrences, and cases with incomplete data were excluded.

The patients underwent ultrasound examination using a linear probe and mammographic imaging in both standard projections—craniocaudal (CC) and mediolateral oblique (MLO). When necessary, further evaluation was performed using tomosynthesis projections, followed by biopsy under either ultrasound or mammographic guidance, with a positive result for breast cancer.

Mammography findings are classified according to the Breast Imaging Reporting and Data System (BI-RADS), and BI-RADS 4 or 5 findings were biopsied to obtain a tissue sample and establish the nature of the lesion. The biopsy was performed in stereotactic mode, i.e., mammography + 3D system, often vacuum-assisted. The selected images, in DICOM format, were completely anonymized during the extraction phase. The presence of microcalcifications was systematically evaluated in the mammography reports by expert breast radiologists.

Breast density classification was extracted from the mammography report performed at the time of diagnosis. A semiquantitative score was used to assess cancer-free breast density and aggregated into four categories using density percentages, similar to the BI-RADS classification: A up to 25%; B 25–50%; C 50–75%; D 75–100%.

Immunohistochemical analyses performed in the pathology department provided data on the expression of estrogen receptor (ER), progestogen receptor (PR), human epidermal growth factor receptor 2 (HER2). In particular, HER2 expression status is currently determined using the American Society of Clinical Oncology/College of American Pathologists (ASCO/CAP) criteria according to which tumors overexpressing HER2 by immunohistochemistry (IHC) 3+ or IHC 2+ with confirmation of HER2 gene amplification by positive in situ hybridization (ISH) are considered “HER2-positive”—tumors with IHC 0, 1+, or 2+ with ISH negativity are considered “HER2-negative”.

Patients were divided into HER2-positive (55) and HER2-negative (130). HER2-positive patients include both luminal B and HER2-enriched.

Tumors were classified into five immunophenotypes based on ER, PR, and HER2 expression: (1) luminal A: ER+/HER2− or PR+/HER2− and Ki-67 proliferation index < 20%; (2) luminal B HER2-negative: ER+/HER2- or PR+/HER2- and Ki-67 proliferation index ≥ 20%; (3) luminal B HER2-positive: ER+/HER2+ or PR+/HER2+ regardless of Ki-67 proliferation index; (4) HER2-enriched: ER−/PR−/HER2+; and (5) triple negative (TN): ER−, PR−, HER2−.

Data were obtained from a review of medical records, radiological reports, and histopathological reports. An anonymized Excel database was created in which the following clinical and pathological data were collected for each patient:

age at diagnosis (dichotomized as ≤50 and >50 years);
presence or absence of microcalcifications detected by digital mammography;
breast composition according to the BI-RADS classification (ACR A, B, C, D);

Histologic tumor type (infiltrating ductal carcinoma [IDC], ductal carcinoma associated with carcinoma in situ [IDC+DCIS], lobular carcinoma [ILC], lobular carcinoma associated with carcinoma in situ [ILC+LCIS], other); molecular subtype determined by immunohistochemistry (luminal A, luminal B HER2 positive, HER2 positive non-luminal, triple negative);

HER2 receptor status assessed by immunohistochemistry (score 0–3+) and, in case of score 2+, confirmed by in situ hybridization (ISH).

Inclusion criteria: women with positive mammography, undergoing biopsy for which the histological result and immunohistochemical study were available.

Exclusion criteria: patients in whom it was not possible to reconstruct the diagnostic–therapeutic path.

### 2.1. Immunohistochemistry (IHC)

IHC is essential to evaluate the expression of the human epidermal growth factor receptor 2 (HER2 receptor), which has an important prognostic and therapeutic value.

A biopsy or surgical specimen of formalin-fixed paraffin-embedded (FFPE) breast tissue is used. The specimen is sectioned into thin slices (approximately 3–5 µm) and mounted on slides. The monoclonal antibody 4B5 (marketed by Ventana/Roche) is applied to the tissue and selectively binds to the HER2 receptor on the membrane of tumor cells. Finally, a chromogenic substrate, usually DAB, is added, which produces a brown stain visible under the microscope in areas where HER2 is present. A counterstain with hematoxylin is performed, which stains the cell nuclei blue to improve contrast and facilitate morphological interpretation. This staining allows the pathologist to visually quantify HER2 expression and assign a diagnostic score (0–3+) according to the ASCO/CAP criteria, adapted to breast cancer.

score 0: no staining or incomplete and weak staining in less than 10% of tumor cells → negative,
score 1+: incomplete and weak staining in more than 10% of tumor cells → negative,
score 2+: weak/moderate and complete staining in at least 10% of cells → equivocal (requires confirmation by FISH),
score 3+: intense and complete staining in more than 10% of tumor cells → positive.

Thus, tumors that overexpress HER2 by immunohistochemistry (IHC) 3+ or IHC 2+ with confirmation of HER2 gene amplification by positive in situ hybridization (ISH) are considered “HER2-positive” tumors with IHC 0, 1+, or 2+ with ISH negativity are considered “HER2-negative”.

The study was conducted in accordance with the principles of the Helsinki Declaration. All data were treated anonymously and no ethics committee approval was required since the study used only retrospective data already available.

### 2.2. Statistical Analysis

The clinical, radiological, and pathological characteristics of the study population were described using descriptive statistics. For each categorical variable included in the model, a reference group was defined for the calculation of odds ratios (ORs): HER2-negative for HER2 status, age ≤ 50 years, ACR A breast density, and histology other than infiltrating ductal carcinoma (IDC). Categorical variables were expressed as absolute frequencies and percentages and compared using the chi-square test or Fisher’s exact test, as appropriate. Continuous variables were reported as mean ± standard deviation or median with interquartile range, depending on the distribution, and compared using the Student t test or Mann–Whitney test.

To identify independent predictors of the presence of mammographic microcalcifications (MCs), a binary logistic regression model was applied. The dependent variable was the presence of MC (yes/no). Independent variables considered in the model included: HER2 status (positive/negative), age (>50 years vs. ≤50 years), breast density (ACR C and D vs. A and B), histological type (IDC vs. others), and molecular subtype (luminal B HER2+). Variables with *p* < 0.10 at univariate analysis were included in the multivariate model.

Results were reported as adjusted odds ratios (aORs) with 95% confidence intervals (95% CIs). A two-tailed *p*-value < 0.05 was considered statistically significant. All statistical analyses were performed using IBM SPSS Statistics^®^, version 26.0 (IBM Corp., Armonk, NY, USA).

We performed both univariate and multivariate analyses to evaluate the association between clinical–radiological variables and the presence of mammographic microcalcifications. Binary logistic regression was used to identify independent predictors. Results are reported as odds ratios (ORs) with 95% confidence intervals (CIs) and corresponding *p*-values. All analyses were performed using IBM SPSS Statistics for Windows, version 26.0 (IBM Corp., Armonk, NY, USA).

## 3. Results

The final study group consisted of 185 women for whom all inclusion criteria were met. A total of 98 women were excluded from the final analysis: 39 due to unknown receptor and molecular subtype, and 59 because the receptor profile was known but the molecular subtype was not.

The analyzed cohort included 185 patients with invasive breast cancer, whose characteristics are shown in Table 1.

Only 55 (29.7%) women had HER2-positive tumors and 130 (70.3%) had HER2-negative tumors. The median age at diagnosis was 61.3 years (range 36–85), with 154 patients (82.3%) older than 50 years.

The distribution of breast density according to the ACR classification was as follows: A (12%), B (45.9%), C (31.4%), and D (9.7%). Analysis of the presence of microcalcifications in the four BI-RADS density categories (ACR A–D) revealed no significant differences between groups. Mammographic microcalcifications were present in 17.4% (4/23) of tumors in ACR A, 18.6% (16/86) in ACR B, 22.4% (13/58) in ACR C, and 16.7% (3/18) in ACR D (chi-square test, *p* = 0.92) (Table 2). Therefore, breast density considered as a four-level variable was not significantly associated with the presence of microcalcifications on mammography. Notably, the higher prevalence of microcalcifications in HER2-positive tumors was consistently observed across all density subgroups. In each ACR category, HER2-positive tumors demonstrated microcalcifications more frequently than HER2-negative tumors (e.g., 33.3% versus 11.8% in ACR A; see Table 2), although these within-category differences did not reach statistical significance due to limited sample sizes in each subgroup.

When breast density was assessed either categorically (ACR A to D) or dichotomously (A–B vs. C–D), no statistically significant association was found with the presence of microcalcifications in either model. This suggests that density alone is not an independent predictor of this radiological feature in the cohort studied. Regarding histological type, 68 cases (37%) were invasive ductal carcinomas (IDCs), 32 (17.3%) IDCs associated with DCIS, 17 (9.2%) invasive lobular carcinomas (ILCs), 5 (3%) ILCs with LCIS, and 63 (34%) other types.

The most represented molecular subtype was luminal A (68%), followed by HER2 positive non-luminal (19.5%), luminal B HER2 positive (10%) and triple negative (2%).

**Figure 1:**
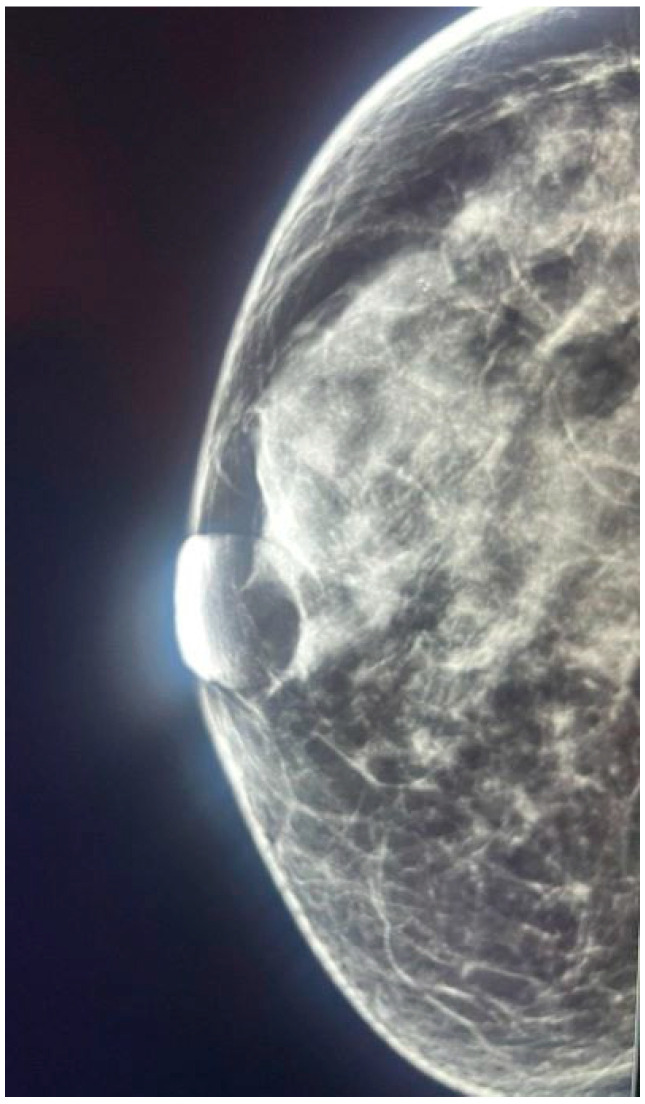
Craniocaudal (CC) mammographic view of a patient with HER2-negative breast cancer (IDC). The image shows a dense breast (BI-RADS C) without suspicious microcalcifications. The absence of suspicious calcification patterns is consistent with the HER2-negative molecular subtype, as observed in the cohort data.

**Figure 2:**
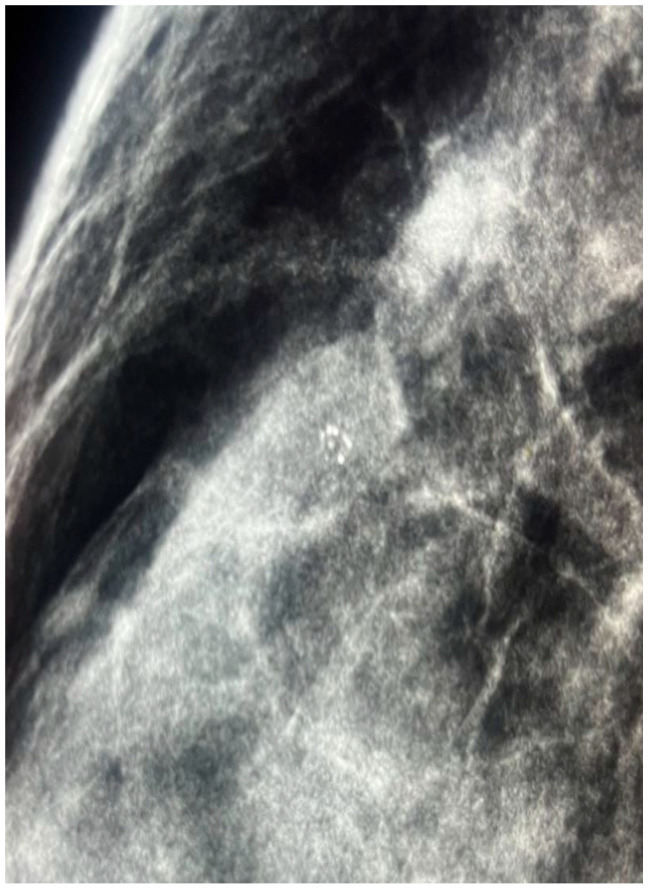
Magnified view from the craniocaudal projection (see Figure 1) showing a cluster of fine, non-suspicious microcalcifications located in the upper outer quadrant (UOQ) of the right breast.

**Figure 3:**
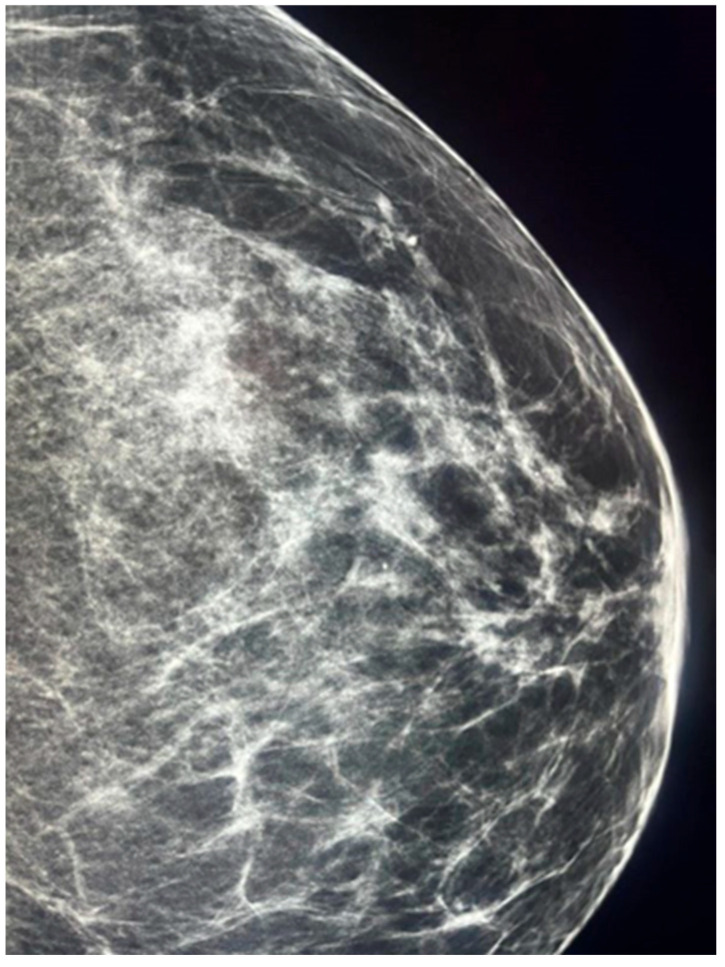
Craniocaudal (CC) mammographic view of the left breast in a patient with invasive ductal carcinoma of no special type (NST). Fine microcalcifications are visible in the periareolar region, displaying a suspicious distribution pattern commonly associated with HER2-positive breast cancer.

**Figure 4:**
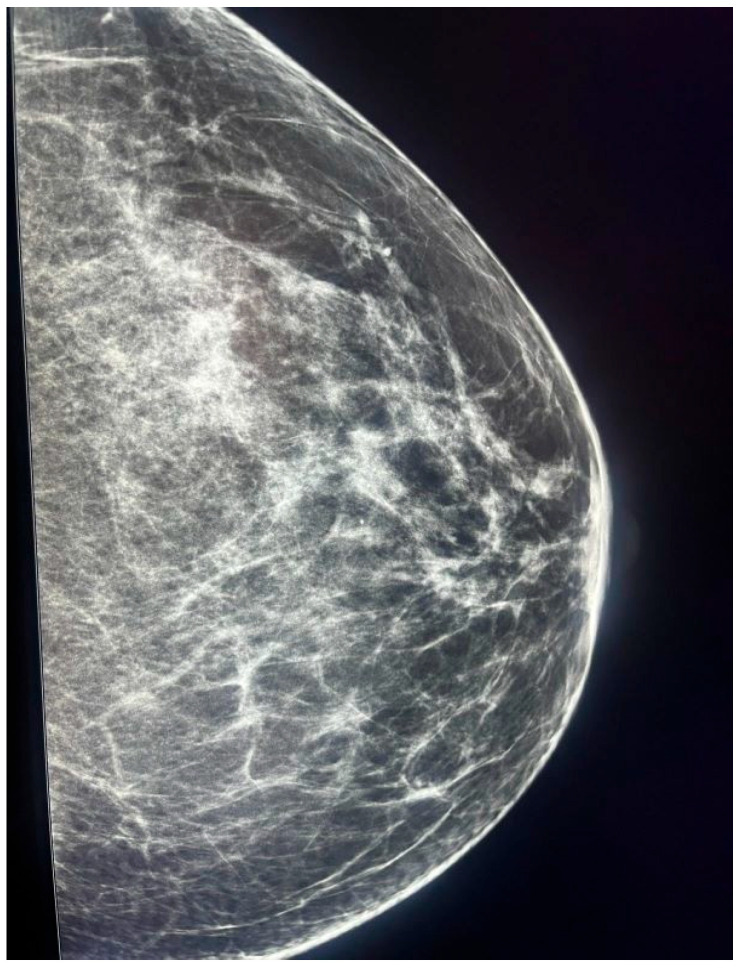
Magnified view of the left periareolar region shown in Figure 3. Fine microcalcifications with segmental distribution and pleomorphic morphology are visible, suggestive of HER2-positive invasive ductal carcinoma.

Mammographic microcalcifications were observed in 36 patients (19.5%), distributed as follows: HER2 positive: 15/55 (27.3%), HER2 negative: 21/130 (16.15%). This difference was statistically significant (*p* < 0.001) (Table 3) (Figure 1, Figure 2, Figure 3 and Figure 4).

In the univariate analysis (Table 4), HER2 positivity (OR 3.08; 95% CI: 1.38–6.87; *p* = 0.006) and the luminal B HER2+ subtype (OR 2.91; 95% CI: 1.02–8.31; *p* = 0.046) were significantly associated with the presence of mammographic microcalcifications. Other clinical–radiological variables, including age > 50 years, breast density (ACR B, C, D), and invasive ductal carcinoma (IDC) histology, did not show statistically significant associations.

In multivariate analysis by binary logistic regression, only HER2-positive status was significantly associated with the presence of microcalcifications, with an adjusted odds ratio of 5.89 (95% CI: 2.42–14.30; *p* < 0.001). None of the other variables (age > 50 years, ACR C or D breast density, IDC histology) showed statistical significance (Table 5).

## 4. Discussion

The results of the present study indicate a statistically significant correlation between HER2 receptor positivity and the presence of microcalcifications on mammography. Microcalcifications, although long considered an early indicator of breast cancer, take on an even more marked predictive value in the presence of overexpression of the HER2 receptor. The literature has highlighted how breast tumors with a prevalence of HER2+ subtypes, are characterized by an unfavorable prognosis and the tendency to metastasize very early [8,9]. In this context, the early adoption of molecular tests in patients with suspected mammography could allow a more rapid identification of candidates for targeted therapy with anti-HER2 drugs, improving the outcome.

These results are consistent with the existing literature. The study by Cen et al. analyzed 485 patients with infiltrating ductal carcinoma, highlighting that BI-RADS 3–5 microcalcifications, particularly those larger than 0.5 mm and extending beyond 2 cm, were significantly associated with the HER2-positive subtype. Multivariate analysis confirmed that these calcification characteristics can be predictive of HER2 positivity [16]. Similarly, Rana et al., in a larger cohort, demonstrated that HER2-positive tumors more frequently present microcalcifications compared to other molecular subtypes, supporting the potential utility of these radiological signs in preliminary molecular stratification [18].

Important is the study by Wang et al. that in a sample of 427 patients with invasive breast cancer, it emerged that “casting” calcifications (linear or branched) were significantly associated with HER2 positivity and hormone receptor negativity. Furthermore, the presence of these calcifications was correlated with a worse prognosis, with lower recurrence-free survival and overall survival rates in HER2-positive patients [19]. Although previous studies have occasionally suggested a link between increased breast density and certain radiological presentations, our analysis, considering density as both a categorical and dichotomous variable, did not reveal any significant relationship with microcalcifications. This indicates that, within our cohort, breast density may not be a confounding or modifying factor in the association between HER2 status and microcalcifications.

Our analysis indicated that breast density (ACR categories A–D) was not significantly associated with the presence of microcalcifications on mammography. This finding suggests that the ability to detect tumor-associated microcalcifications was not substantially impaired in patients with higher breast density. Indeed, although it is well documented that the overall sensitivity of mammography for breast cancer detection decreases with increasing breast density—from approximately 86–89% in fatty breasts (ACR A) to approximately 62–68% in extremely dense breasts (ACR D)—mammography remains effective in identifying microcalcifications even in dense tissue [20]. Microcalcifications appear as high-contrast spots on radiographic images, so they can often be visualized despite the radiographic masking effect of dense fibroglandular tissue. Clinically, this is relevant because it means that in women with dense breasts (ACR C or D), lesions that manifest with microcalcifications (such as some DCIS or HER2-overexpressing tumors) may still be detected by mammography, whereas noncalcified masses are more likely to be missed in dense tissue. However, the overall reduced sensitivity in dense breasts means that a normal mammogram does not rule out cancer in these patients and that additional imaging modalities (such as tomosynthesis, ultrasound, or MRI) may be considered to improve detection in ACR C and D breasts. The consistent trend we observed—higher rates of microcalcification in HER2-positive tumors across all density categories—further underscores that the association between HER2 overexpression and microcalcifications is robust, regardless of breast composition. This resilience in detecting microcalcifications across all densities supports the reliability of mammography for identifying HER2-positive tumors, even in patients with dense breast parenchyma. However, it remains critical that radiologists be aware of the limitations imposed by breast density and maintain a high index of suspicion when interpreting mammograms of patients with heterogeneously or extremely dense breasts.

This study is distinguished by the use of multivariate logistic regression, which allows the HER2 variable to be assigned an independent effect relative to other clinical characteristics. Neither age over 50 years nor high breast density (ACR C or D) nor histological type (IDC) proved to be significant predictive factors. This suggests that the observed correlation between microcalcifications and HER2 is not due to bias related to the population’s demographic data but likely reflects a biological interaction between the tumor genotype and the radiologic phenotype.

All of this suggests the existence of a shared biological pattern between HER2 expression and microcalcifications, regardless of histopathological classification or clinical presentation.

Studies had already associated microcalcifications with HER2+ and high-grade tumors [21,22,23]. However, the novelty of recent years is represented by experimental works that reveal an active role of calcifications in promoting EMT, chronic inflammation and aberrant mineralization [24].

Breast microcalcifications, frequently observed in HER2-positive tumors, are not simple radiological findings but reflect complex biological alterations. In HER2+ carcinomas, rapid cell proliferation leads to central necrosis and subsequent dystrophic calcification, particularly in high-grade ductal carcinoma in situ with a comedonic pattern [25,26]. However, recent studies demonstrate that HER2+ tumor cells can acquire an osteoblastic phenotype, actively producing hydroxyapatite crystals through activation of epithelial-mesenchymal transition (EMT) and the expression of genes such as BMP-2, RUNX2, and osteopontin [20]. Furthermore, microcalcifications can recruit tumor macrophages (TAMs) that release osteogenic and pro-inflammatory factors (e.g., IL-1β), promoting tumor invasiveness [27,28]. This creates a pro-tumor microenvironment that facilitates neoplastic progression, suggesting an active role of calcifications in tumor biology [29].

This evidence strengthens the importance of the combined use of imaging diagnostics and molecular profiling in the early stratification of cancer risk, contributing to the identification of patient subgroups who may benefit from targeted anti-HER2 therapies.

Microcalcifications, especially linear or segmental ones, are an early marker of HER2+ DCIS and guide the indication for stereotactic biopsy. In the preoperative phase, their distribution may influence the choice between conservative surgery or mastectomy, especially in multifocal cases. In HER2+ tumors undergoing neoadjuvant chemotherapy, the persistence of microcalcifications raises questions: they often represent residual DCIS, which must be surgically removed, even in the absence of residual invasive disease [30]. Therefore, the calcific pattern influences therapeutic, surgical, and follow-up decisions.

In addition, the application of radiomics has allowed the extraction of quantitative parameters from mammographic images to accurately predict HER2 status (AUC~0.85), anticipating molecular information before biopsy [31]. Artificial intelligence can now identify suspicious patterns (linear clusters, casting-type) and suggest a probable HER2+ phenotype, with possible implications in patient stratification and planning of targeted treatments, such as anti-HER2 therapies or extensive surgery. These approaches, although still in validation, represent the future of radiogenomic and precision medicine.

Strengths include the fact that the study is based on a well-characterized cohort of patients with nonpalpable breast cancer, with complete data on imaging, histology, and molecular profile. The integration of mammographic findings and immunohistochemical assessment allowed a multidimensional analysis of the relationship between microcalcifications and HER2 status. The use of adequate statistical tools, including exact tests and odds ratios with confidence intervals and the use of multivariate logistic regression, ensured the robustness of the results.

The study has some limitations. It is a single-center retrospective analysis with a relatively small number of HER2-positive patients with mammography. Additionally, mammographic imaging was assessed based on preexisting reports and not by central review. The data relating to microcalcifications were obtained from the original imaging reports without independent rereading or direct correlation with pathological anatomy, which could introduce interobserver variability. Moreover, the lack of inclusion of relevant morphological and clinical variables—such as tumor size, histological grade, and mammographic pattern of microcalcifications—represents a limitation of the predictive model, reducing its accuracy and applicability. Future studies should incorporate these parameters together with radiomics and artificial intelligence tools to build more accurate predictive models of HER2 status.

Further prospective multicenter studies with standardized radiological review could strengthen the evidence for these findings.

## 5. Conclusions

Microcalcifications on mammography are significantly associated with HER2 positivity in breast cancer. These findings could represent a useful alarm signal, especially in screening settings, and justify early molecular characterization. The integration of imaging and molecular biology may improve early diagnosis and personalized management of patients.

## Figures and Tables

**Table 1:** Clinical and pathological characteristics of the analyzed cohort.

Characteristic	Absolute Value	Percentage (%)
Total number of patients	185.0	
Mean age (years)	61.3	
Age > 50 years	154.0	83.2
**Breast Density**		
Breast density ACR A	23.0	12.4
Breast density ACR B	86.0	46.5
Breast density ACR C	58.0	31.4
Breast density ACR D	18.0	9.7
**Histology**		
Invasive Ductal Carcinoma (IDC)	68.0	36.8
Invasive Ductal Carcinoma (IDC) + Ductal Carcinoma In Situ (DCIS)	32.0	17.3
ILC histology	17.0	9.2
ILC + LCIS histology	5.0	2.7
Other histologies	63.0	34.0
**Molecular type**		
Luminal A	126.0	68.1
Luminal B HER2+	19.0	10.3
HER2+ (non-luminal)	36.0	19.5
Triple-negative	4.0	2.2
**Microcalcifications**		
Total microcalcifications	36.0	19.5
Microcalcifications in HER2+	15.0	27.3
Microcalcifications in HER2−	21.0	16.15

This table shows the distribution of clinical variables (age, breast density according to BI-RADS ACR) and pathological variables (histotype, molecular subtype) of the 185 patients included in the study. The percentages are calculated with respect to the total. Abbreviations: ACR = American College of Radiology (breast density classification); IDC = invasive ductal carcinoma; DCIS = ductal carcinoma in situ; ILC = invasive lobular carcinoma; LCIS = lobular carcinoma in situ; HER2 = human epidermal growth factor receptor 2.

**Table 2:** Mammographic microcalcifications in relation to BI-RADS breast density (ACR categories A–D) and HER2 status.

ACR Density	HER2-Positive Tumors, N°(with Microcalcifications N°, %)	HER2-Negative Tumors, N° (with Microcalcifications N°, %)
A (almost entirely fatty)	6 (2, 33.3%)	17 (2, 11.8%)
B (scattered fibroglandular)	25 (6, 24.0%)	61 (10, 16.4%)
C (heterogeneously dense)	17 (5, 29.4%)	41 (8, 19.5%)
D (extremely dense)	7 (2, 28.6%)	11 (1, 9.1%)

Data are expressed as number of tumors, with the number and percentage of those tumors showing microcalcifications in parentheses.

**Table 3:** Distribution of microcalcifications according to breast cancer molecular subtypes.

Molecular Subtype	N°	Percentage	Microcalcifications n°
Luminal A	126	68%	21
Luminal B HER2+	19	10%	8
Luminal B HER2−	0	0%	0
HER2+	36	19%	7
Triple Negative	4	2%	0

This table shows the number and percentage of microcalcifications detected for each molecular subtype of breast cancer. Subtypes were defined based on the expression of ER, PR, and HER2, according to immunohistochemical classification. The data suggest a higher frequency of microcalcifications in HER2-positive tumors (both luminal B HER2+ and HER2-enriched) compared to other subtypes.

**Table 4:** Univariate analysis: association between clinical–radiological variables and presence of microcalcifications.

Variable	Raw OR	CI 95% (Low–High)	*p*-Value
Age > 50 years old	1.37	0.51–3.71	0.529
Breast Density ACR B	1.15	0.42–3.15	0.779
Breast Density ACR C	1.72	0.64–4.59	0.280
Breast Density ACR D	1.08	0.27–4.39	0.916
IDC	0.79	0.34–1.85	0.589
HER2-positive	3.08	1.38–6.87	0.006
Luminal B HER2+	2.91	1.02–8.31	0.046

Univariate analysis evaluates the correlation between selected variables and the presence of microcalcifications on mammography. Odds ratios (ORs), confidence intervals (95% CIs), and *p* values calculated by simple logistic regression are reported. Abbreviations: Raw OR = unadjusted odds ratio; CI = confidence interval.

**Table 5:** Multivariate analysis (logistic regression): independent predictors of microcalcifications.

Variable	Adjusted OR	Inferior IC 95%	Superior IC 95%	*p*-Value
Positive HER2	5.89	2.42	14.30	<0.001
Age > 50 years	1.41	0.43	4.65	0.570
Density ACR C	1.35	0.54	3.38	0.520
Density ACR D	0.88	0.16	4.86	0.886
Ductal histology (IDC)	0.69	0.29	1.64	0.397

Note: Adjusted OR: odds ratio adjusted with multiple covariates. The reference groups for OR calculation are as follows: HER2-negative for HER2 status; age ≤ 50 years; ACR A breast density; histology other than infiltrating ductal carcinoma (IDC). The multivariate model included significant or potentially confounding variables. The analysis confirmed that HER2-positive status was an independent predictor of microcalcifications. The table shows the adjusted odds ratios (ORs) and the corresponding 95% confidence intervals (95% CIs) for the factors examined in the multivariate analysis of the presence of microcalcifications on mammography. Only HER2-positive status showed a statistically significant association with the presence of microcalcifications (OR = 5.89; 95% CI: 2.42–14.30; *p* < 0.001), while age > 50 years, high breast density (ACR categories C and D), and invasive ductal histology (IDC) were not significantly associated with such presence (*p* > 0.05 for all). Abbreviations: OR = odds ratio; CI = confidence interval; HER2 = human epidermal growth factor receptor 2; ACR = American College of Radiology (breast density classes); IDC = invasive ductal carcinoma.

## Data Availability

The data are contained in the article.
